# Development, Interlaboratory Evaluations, and Application of a Simple, High-Throughput *Shigella* Serum Bactericidal Assay

**DOI:** 10.1128/mSphere.00146-18

**Published:** 2018-06-13

**Authors:** Moon H. Nahm, Jigui Yu, Hailey P. Weerts, Heather Wenzel, Chitradevi S. Tamilselvi, Lakshmi Chandrasekaran, Marcela F. Pasetti, Sachin Mani, Robert W. Kaminski

**Affiliations:** a University of Alabama at Birmingham, Birmingham, Alabama, USA; b Department of Enteric Infections, Bacterial Diseases Branch, Walter Reed Army Institute of Research, Silver Spring, Maryland, USA; c PATH, Washington, DC, USA; d Center for Vaccine Development, University of Maryland, Baltimore, Maryland, USA; Parasitology Services

**Keywords:** *Shigella*, antibody, functional assay, immunoassay

## Abstract

*Shigella* is an important cause of diarrhea worldwide, with serotypes Shigella flexneri 2a, S. flexneri 3a, and Shigella sonnei demonstrating epidemiological prevalence. Many development efforts are focused on *Shigella* lipopolysaccharide (LPS)-based vaccines, as O antigen-specific conjugate vaccines are immunogenic and efficacious. Immunization with *Shigella* vaccines containing LPS can elicit antibodies capable of killing *Shigella* in a serotype-specific manner. Thus, to facilitate *Shigella* vaccine development, we have developed a serum bactericidal assay (SBA) specific for three *Shigella* serotypes that measures killing of target bacteria at multiple serum dilutions and in the presence of exogenous complement. The SBA has a high analytical throughput and uses simple technologies and readily available reagents. The SBA was characterized with human sera with bactericidal antibodies against S. flexneri 2a, S. flexneri 3a, and S. sonnei. Purified LPS of a homologous serotype, but not a heterologous serotype, inhibited bacterial killing. Assessment of precision found median intra-assay precision to be 13.3% and median interassay precision to be 19 to 30% for the three serotypes. The SBA is linear, with slight deviations for samples with low (~40) killing indices. The SBA was sensitive enough to allow about 100-fold predilution of serum samples. Repeat assays yielded results with less than 2-fold deviations, indicating the robustness of the assay. Assay results from four different laboratories were highly comparable when normalized with a reference serum. The *Shigella* SBA, combined with a reference serum, should facilitate the development of *Shigella* vaccines across the field.

**IMPORTANCE** *Shigella* is an important cause of diarrhea worldwide, and efforts are ongoing to produce a safe and effective *Shigella* vaccine. Although a clear immune correlate of protection has not been established, antibodies with bactericidal capacity may provide one means of protecting against shigellosis. Thus, it is important to measure the functional capacity of antibodies, as opposed to only binding activity. This article describes a simple, robust, and high-throughput serum bactericidal assay capable of measuring *Shigella*-specific functional antibodies *in vitro*. We show for the first time that this assay was successfully performed by multiple laboratories and generated highly comparable results, particularly when SBA titers were normalized using a reference standard. The serum bactericidal assay, along with a reference serum, should greatly facilitate *Shigella* vaccine development.

## Abstract (precis)

*Shigella* is an important cause of diarrhea worldwide, and efforts are ongoing to produce a safe and effective *Shigella* vaccine. Although a clear immune correlate of protection has not been established, antibodies with bactericidal capacity may provide one means of protecting against shigellosis. Thus, it is important to measure the functional capacity of antibodies, as opposed to only binding activity. This article describes a simple, robust, and high-throughput serum bactericidal assay capable of measuring *Shigella*-specific functional antibodies *in vitro*. We show for the first time that this assay was successfully performed by multiple laboratories and generated highly comparable results, particularly when SBA titers were normalized using a reference standard. The serum bactericidal assay, along with a reference serum, should greatly facilitate *Shigella* vaccine development.

## INTRODUCTION

*Shigella* remains, according to the Global Enteric Multicenter Study (GEMS), one of the top five attributable agents of moderate-to-severe diarrhea among children less than 5 years of age living in sub-Saharan Africa and South Asia (1). It has also been associated with sporadic outbreaks and epidemics worldwide, resulting in significant morbidity and mortality (2). In addition, travelers and military personnel represent other high-risk groups (3).

There are four different species of *Shigella*—Shigella sonnei, Shigella flexneri, Shigella boydii, and Shigella dysenteriae. Each species can be further divided into multiple serotypes based on the immunologic properties of the O antigen part of lipopolysaccharide (LPS). For instance, S. flexneri has 14 different serotypes with unique LPS O antigen structures (4). Knowledge of species and serotypes is important because there is little cross-protective immunity among different serotypes and they differ in geographic distribution. S. dysenteriae and S. boydii are rare in the United States, but S. sonnei and S. flexneri are common in the United States and throughout the world. Three of the most relevant *Shigella* serotypes are S. flexneri 2a, S. flexneri 3a, and S. sonnei, which are major causes of morbidity and mortality in the developing world (5).

*Shigella* vaccines are highly desirable as a public health control measure, especially in view of the emergence of drug-resistant strains (6). At present, several different approaches are used to produce *Shigella* vaccines. One of the main strategies targets the serotype-specific LPS antigen, for several reasons. LPS has been identified as a key antigen recognized by the immune system after natural infection (7, 8), and LPS antibody production has been correlated with protection from homologous serotypes (8–11). Also, successful experiences with multivalent pneumococcal conjugate vaccines (12), combined with the successes of *Shigella* conjugate vaccines targeting the O antigen (13), suggest that a multivalent *Shigella* conjugate vaccine could induce antibodies to LPS O antigens from multiple serotypes to confer broad protection.

Although the human immune system provides protection against *Shigella* via multiple mechanisms that are not yet fully elucidated, antibodies to LPS O antigens can fix complement and kill target bacteria in a serotype-specific manner (14, 28). Thus, a serum bactericidal assay (SBA) that replicates this killing process *in vitro* would allow the detection of antibodies that could display bactericidal activity *in vivo* and contribute to protective immunity. A recent study reported a strong association between S. flexneri 2a-specific SBA titers in human adult volunteers and reduced clinical disease postchallenge with wild-type organisms (15), thus supporting the value of this assay to potentially predict vaccine efficacy.

Important features that would make an SBA useful to facilitate vaccine development include high analytical throughput to handle many samples, robustness, reproducibility, and ease of performance, so that it could be applied by multiple laboratories. Here, we describe the establishment and qualification of a high-throughput and robust SBA for S. flexneri 2a, S. flexneri 3a, and S. sonnei and present results from an interlaboratory assay evaluation.

## RESULTS

### Development of a high-throughput *Shigella* SBA.

We had previously transformed a classical pneumococcal opsonophagocytosis assay and *Haemophilus* SBA into high-throughput assays by using cryopreserved target bacteria and by automating colony counting (17, 18). The same strategies were applied to the development of a *Shigella* SBA. First, we found that *Shigella* strains, including S. flexneri 2a strain 2457T, S. flexneri 3a strain J17B, and S. sonnei strain Moseley (Table 1), could be aliquoted, stored at −80°C, and thawed after more than 1 year while still maintaining high viability (M.H.N., personal observation). The use of cryopreserved target strains eliminates the time-consuming bacterial culture step required on the day of assay and reduces variability. Next, we optimized a methodology to produce miniature (~0.2 mm in diameter) *Shigella* colonies with color. Triphenyl tetrazolium chloride (TTC) in an agar overlay containing 0.1% sodium azide is used to color metabolically active *Shigella* colonies deeply red. Because *Shigella* colonies often become too large after overnight incubation at 37°C, we reduced their size with overnight incubation at lower temperatures, 29°C for strains S. flexneri 2a 2457 T and S. flexneri 3a J17B and 26°C for S. sonnei Moseley. The colony miniaturization permits one agar plate to be subdivided into 48 subregions, each of which can accommodate about 160 distinct microcolonies (see Fig. S1 in the supplemental material). Thus, two agar plates can accommodate the equivalent of a 96-well microtiter plate (2 × 48 subregions). This dramatic reduction in agar plate numbers eliminates the bottleneck created by the logistical requirements and labor intensivenes of handling numerous agar plates.

10.1128/mSphere.00146-18.1 FIG S1  An example of SBA results. Left panel shows LB agar plates that show bacterial colonies from eight heat-inactivated complement controls (first column from the left, marked CTL-A at the top), eight active complement controls (second column, marked CTL-B at the top), and SBA results of two MAbs, Hflex2a1 and Hflex2a4. Rows 1 through 8 indicate 3-fold serial dilutions of the MAbs from 1:4 (row 1) to 1:8,748 (row 8). Hflex2a4 was prediluted at 1:5,000. Right panel is a graph showing colony counts (*y* axis) at different serum dilutions in an assay plate (*x* axis). Average CFU of CTL-B wells are used to determine 0% killing (solid line) and 50% killing (dashed line). Download FIG S1, DOCX file, 0.6 MB. Copyright © 2018 Nahm et al. 2018 Nahm et al. This content is distributed under the terms of the Creative Commons Attribution 4.0 International license.

**TABLE 1:** Bacterial strains used in the study

*Shigella* serotype	Strain	Source
S. dysenteriae 1	ATCC 9361	ATCC
S. dysenteriae 2	ATCC 9750	ATCC
S. flexneri 2a	2457T	WRAIR
S. flexneri 2a	MHK03875	WRAIR
S. flexneri 2b	ATCC 12022	ATCC
S. flexneri 3a	J17B	WRAIR
S. flexneri 3a	MHK04508	WRAIR
S. boydii 1	ATCC 9207	ATCC
S. sonnei	Moseley	WRAIR
S. sonnei	53G	WRAIR

### Optimization of SBA conditions.

**FIG 1:**
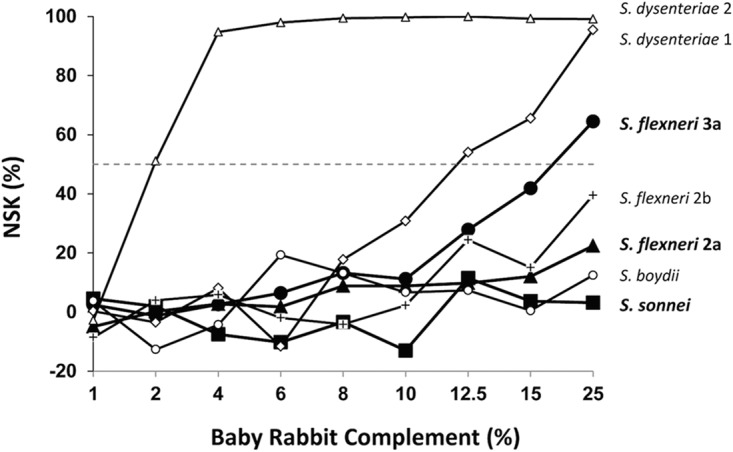
Nonspecific killing (NSK) of various bacterial strains at different concentrations (%) of baby rabbit complement after 2-h incubation at 37°C in room air. The strains are 2457T for S. flexneri 2a, J17B for S. flexneri 3a, Moseley for S. sonnei, ATCC 9361 for S. dysenteriae 1, ATCC 9750 for S. dysenteriae 2, ATCC 12022 for S. flexneri 2b, and ATCC 9207 for S. boydii. Dotted horizontal line indicates 50% killing.

Various assay parameters were optimized to reduce the potential impact of method deviations on assay results. First, we tested different lots and concentrations of baby rabbit complement (BRC) using multiple *Shigella* strains to minimize nonspecific killing (NSK). When strains were incubated with low (<8%) BRC concentrations for 2 h at 37°C without supplemented CO_2_, all strains showed very low NSK (Fig. 1). However, if less than 10% BRC is used, the alternative pathway becomes inactive (19) and cannot amplify complement activation, and the SBA may become insensitive. When 15% BRC was used, all three relevant serotypes (S. flexneri 2a, S. flexneri 3a, and S. sonnei) showed less than 50% NSK. This level of NSK has also been found to be acceptable in the case of the pneumococcal opsonophagocytic killing assay (OPA) (M.H.N., personal observation). Thus, a BRC concentration of 12.5% (midpoint between 10% and 15%) was chosen for the SBA.

**FIG 2:**
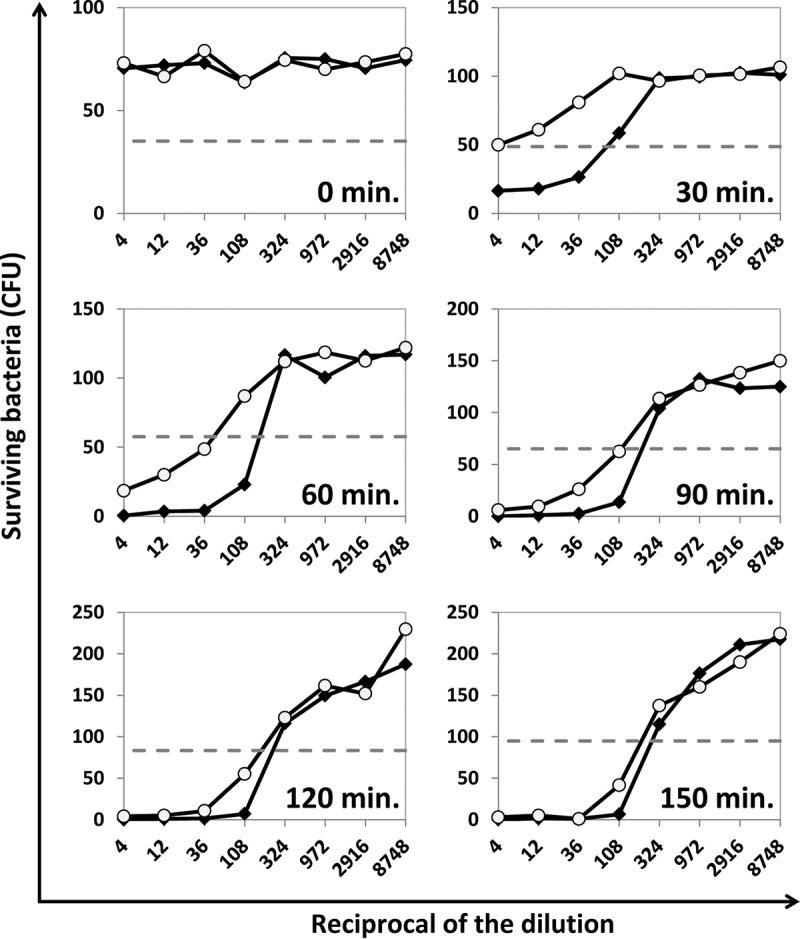
Evaluation of SBA incubation times for S. flexneri 2a. The numbers of surviving S. flexneri 2a colonies (*y* axis) after incubating with serially diluted MAb Hflex2a1 (solid diamonds) or human serum pool 007sp (open circles) (*x* axis) are shown at various time points. Dashed horizontal line indicates 50% killing. CFU counts obtained with control wells were used as 0% killing.

The SBA has an incubation step at 37°C for complement activation and killing of target bacteria, but this incubation temperature also supports bacterial replication. To investigate the two opposing aspects, we studied various reaction times (30 min to 150 min) utilizing serotype-specific monoclonal antibodies (MAbs) and 007sp, a human serum pool made by the U.S. FDA that is used as a standard for pneumococcal antibody assays (20). When using S. flexneri 2a as the target bacterium and short reaction times, such as 30 min, only partial killing was observed even at high antibody levels (Fig. 2). However, longer reaction times, such as 90, 120, and 150 min, completely killed bacteria at higher antibody concentrations and showed a sigmoidal survival curve as antibody levels decreased (Fig. 2). Dose-response curves were demonstrated despite bacterial replication of approximately 2.5-fold during the assay period (average CFU counts for the active complement control wells increased from ~80 to ~200 CFU/spot over the course of incubation) (Fig. 2). Similar findings were observed for S. flexneri 3a and S. sonnei (Fig. S2). Thus, a 120-min incubation was chosen for this SBA.

10.1128/mSphere.00146-18.2 FIG S2  SBA performed with various incubation times. The numbers of surviving bacterial colonies (*y* axis) at various dilutions of MAbs (solid diamonds) or a human serum pool (007sp) (open circles) (*x* axis) are shown. The incubation times used for SBA are indicated in minutes in the lower right corner. (A) The experiment whose results are shown used target strain J17B for S. flexneri 3a and MAb Hflex3a2. (B) The experiment whose results are shown used target strain Moseley for S. sonnei and MAb Hsoni1. Download FIG S2, DOCX file, 0.2 MB. Copyright © 2018 Nahm et al. 2018 Nahm et al. This content is distributed under the terms of the Creative Commons Attribution 4.0 International license.

### Selection of the target bacterial strains.

**FIG 3:**
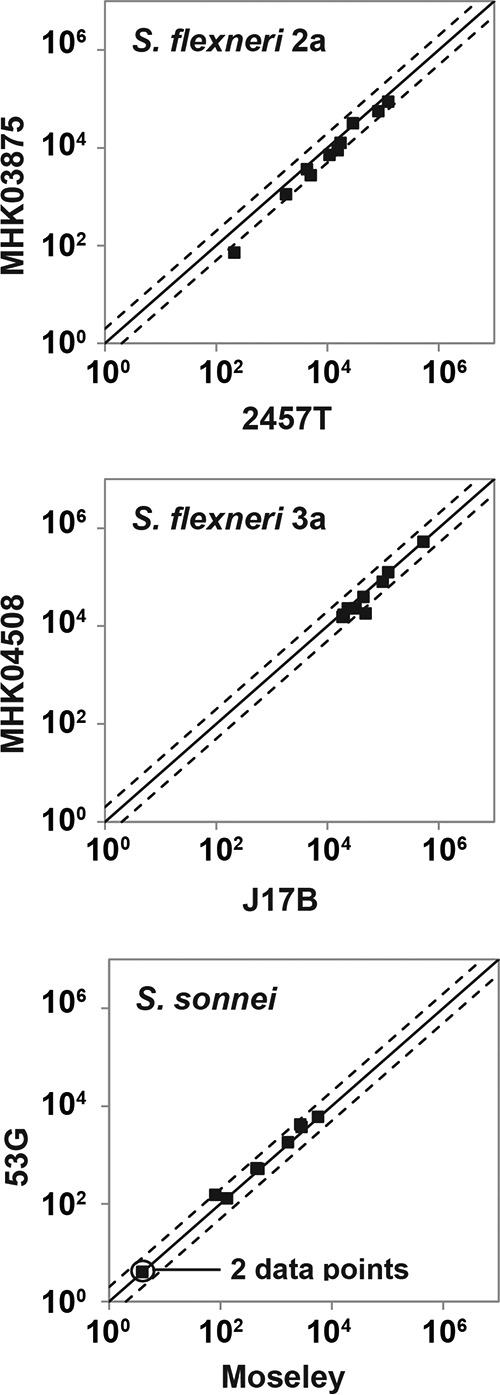
Comparison of killing indices obtained with different SBA target strains. The SBA was performed with two different target strains indicated in *x* and *y* axes using 10 serum samples from healthy adults. The line of identity (solid line) and a 2-fold deviation from the line of identity (dotted lines) is shown. Pearson’s *R*^2^ is 0.98 for S. flexneri 2a, 0.93 for S. flexneri 3a, and 0.99 for S. sonnei.

S. flexneri 2a, 2457T, S. flexneri 3a, J17B, and S. sonnei, Moseley, were chosen as target strains because they are clinically relevant and widely used in vaccine development and preclinical and clinical efficacy studies (21, 22). Alternative target strains (of the same serotype) were also evaluated to ensure that the SBA would generate consistent results. The bactericidal activities of the LPS-specific MAbs were comparable (*P* values of ≥0.363 and correlation coefficients of ≥0.994; paired *t* test) when tested against two different target bacterial strains sharing the same LPS serotypes (Table 2). We then compared the target bacterial strains with 10 individual human sera. The strains sharing LPS serotypes yielded comparable SBA killing indices (KIs) (*r*^2^ > 0.93 for all three serotypes) (Fig. 3), but strains of different serotypes produced disparate KIs (Fig. S3). These results also confirm the presence of naturally acquired bactericidal antibodies in adults, which mostly target *Shigella* LPS O antigen (7, 8).

10.1128/mSphere.00146-18.3 FIG S3  Comparison of different target strains for SBA. The SBA was performed with two different strains, indicated on *x* and *y* axes, using 10 serum samples from healthy adults. LPS serotypes are indicated in parentheses next to the strain names. The line of identity (solid line) is shown along with 2-fold deviations (dotted lines). Data points are along the identity lines only when the two serotypes are matched. Download FIG S3, DOCX file, 0.3 MB. Copyright © 2018 Nahm et al. 2018 Nahm et al. This content is distributed under the terms of the Creative Commons Attribution 4.0 International license.

**TABLE 2:** Bactericidal killing indices of *Shigella* serotype-specific MAbs against several reference strains of *Shigella* spp.

MAb	KI of indicated strain					
	S. flexneri 2a		S. flexneri 3a		S. sonnei	
	MHK03875	2457T	MHK04508	J17B	53G	Moseley
Hflex2a1	900	1,032	<4	<4	<4	<4
Hflex2a4	489,428	722,794	<4	<4	<4	<4
Hflex3a2	<4	<4	1,933	2,913	<4	<4
Hflex3a5	<4	<4	3,309,914	2,771,795	<4	<4
Hsoni1	<4	<4	<4	<4	728	630
Hsoni5	<4	<4	<4	<4	6,235	2,774

### Bactericidal activity of normal unvaccinated sera is mostly directed against LPS O antigen.

**FIG 4:**
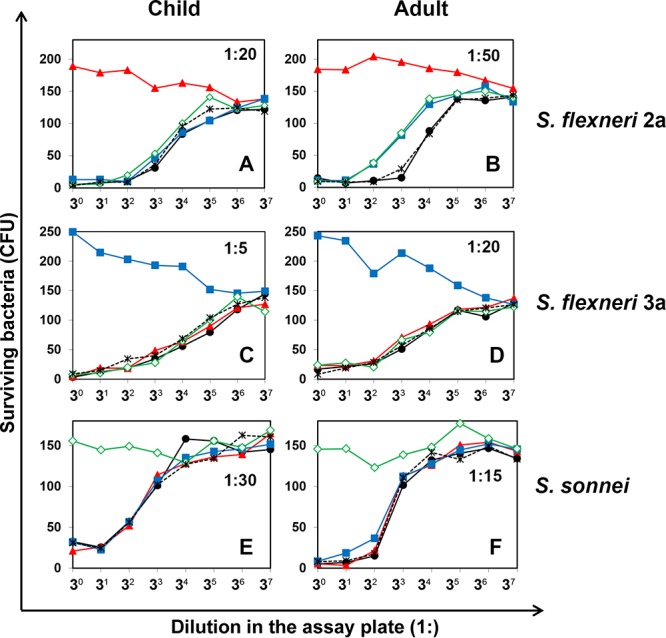
Impact of LPS on bactericidal activities of serum samples from a child (A, C, and E) and an adult (B, D, and F). LPS was from S. flexneri 2a (solid triangles, red), S. flexneri 3a (solid squares, blue), S. sonnei (open diamonds, green), and E. coli (stars, black). LPS-free buffer (solid circles, black) was also used as a negative control. A prediluted serum was mixed with LPS (final LPS concentration, 25 µg/ml) and incubated for 10 min at room temperature before it was used for the SBA. Serum samples were prediluted 1:20 (A), 1:50 (B), 1:5 (C), 1:20 (D), 1:30 (E), and 1:15 (F). Serotypes of SBA target strains are S. flexneri 2a (A and B), S. flexneri 3a (C and D), and S. sonnei (E and F).

To test whether the bactericidal activity of normal serum targets LPS, we examined the effect of free LPS on the bactericidal activity of a serum from a healthy child (Fig. 4A, C, and E) and an adult serum (Fig. 4B, D, and F). LPS (50 µg/ml) from S. flexneri 2a, S. flexneri 3a, S. sonnei, or Escherichia coli was mixed with an equal volume of a prediluted serum (predilutions are described in the legend to Fig. 4). When the SBA was performed with the serum-LPS mixtures, the bactericidal killing against homologous LPS was completely blocked and serum-facilitated bacterial replication during assay incubation was permitted (Fig. 4). Preincubation with LPS from heterologous bacterial strains did not interfere with killing and, thus, had no detectable effect on the SBA titers for S. flexneri 3a and S. sonnei. However, in the case of the S. flexneri 2a SBA, purified LPS from S. flexneri 3a and S. sonnei reduced the killing activity by about 3-fold, whereas E. coli LPS did not reduce killing (Fig. 4B), suggesting that LPS from heterologous *Shigella* serotypes may partially inhibit the bactericidal activity of antibodies. Collectively, the bactericidal activity of normal sera appears to be largely due to antibodies to O antigen of LPS. However, normal sera may contain antibodies capable of killing S. flexneri 2a by targeting other antigens, and this possibility should be further investigated.

### Linearity of the SBA.

**FIG 5:**
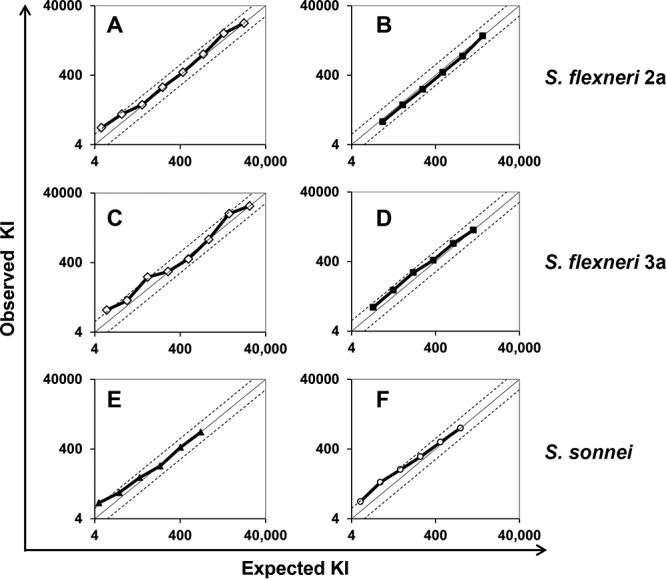
Observed KIs (*y* axis) were compared to expected KIs (*x* axis) for each sample. Expected KI was calculated by dividing the KI of the undiluted sample by the predilution factors. Two different adult serum samples were used for S. flexneri 2a (A and B), S. flexneri 3a (C and D), and S. sonnei (E and F). The line of identity (solid line) is shown along with two dotted lines showing 2-fold deviations from the identity.

Assay linearity was assessed by utilizing two sets of serum samples representing a wide range of SBA activities (from low to high). To prepare such samples, two human sera with high bactericidal activity were 3-fold serially diluted with a serum exhibiting no bactericidal activity, which is commercially available for this purpose (special stripped serum; Valley Biomedical, Winchester, VA). Expected KIs were calculated by dividing the KI of the sample before serial dilution by the dilution factor. Then, all the samples made from the two original sera were analyzed by SBA and their KIs were experimentally determined. When the observed and expected KIs were plotted, comparable levels of bactericidal activity were demonstrated, except at lower KI values, but remained within about a 2-fold range of deviations (Fig. 5). The lower limit of quantitation was determined to be a KI of between 10 and 40 for each of the three serotypes. However, considering that the accepted range of variations for most functional assays is 2-fold (23), the observed nonlinearity at low KIs may be insignificant.

### Intra- and interassay precision of SBA.

The assay precision (coefficient of variation [CV]) was determined with a panel of human sera using target bacteria and one commercial lot of complement. Intra-assay precision, or within-run precision, was determined by testing three serum samples from healthy adults, one serum sample from a child, and one MAb, each run 10 times in one assay. The intra-assay CV ranged from 5.8 to 23.1% (Table 3), with a median CV of 13.3% for all three serotypes. Interassay precision was determined by analyzing eight well-characterized adult serum samples 10 times on different days over a 3-week interval (Table 4). While one serum sample (SS10) produced high CVs (~60%) for both S. flexneri 2a and S. flexneri 3a assays for unknown reasons, the CVs for all other samples were below 45% and the median values of the CVs ranged from 18 to 28% for the three serotypes.

**TABLE 3:** Intra-assay precision

Serotype	Sample	Mean SBA titer	SD	CV (%)
S. flexneri 2a	Hflex2a1	279	28	9.9
	SS2^a^	14,065	1,794	12.8
	SS3	49,505	11,416	23.1
	SS4	7,129	663	9.3
	SS5	7,392	1,321	17.9
S. flexneri 3a	Hflex3a2	2,565	314	12.2
	SS6^a^	42,430	5,688	13.4
	SS3	30,586	6,848	22.4
	SS4	18,563	4,052	21.8
	SS5	17,734	2,522	14.2
S. sonnei	Hsoni1	672	49	7.3
	SS7^a^	14,061	1,874	13.3
	SS3	4,953	447	9
	SS4	480	28	5.8
	SS5	474	70	14.8

a Sample was from a child.

**TABLE 4:** Interassay precision

Serotype	Sample	Mean SBA titer	SD	CV (%)
S. flexneri 2a	SS8	12,050	3,710	30.8
	SS9	25,886	5,868	22.7
	SS10	89,165	58,104	65.2
	SS11	153,218	64,465	42.1
	SS12	573	245	42.7
	SS13	16,769	3,785	22.6
	SS4	7,709	1,994	25.9
	SS5	8,126	1,481	18.2
S. flexneri 3a	SS8	19,264	6,928	36
	SS9	28,485	7,483	26.3
	SS10	66,678	40,581	60.9
	SS11	39,219	7,558	19.3
	SS12	292,901	81,875	28
	SS13	43,189	11,136	25.8
	SS4	14,822	2,873	19.4
	SS5	14,668	3,120	21.3
S. sonnei	SS3	5,170	1,586	30.7
	SS14	1,694	330	19.5
	SS15	1,276	501	39.3
	SS11	92	10	11.4
	SS4	406	26	6.4
	SS5	449	55	12.3
	SS16	1,189	207	17.4
	SS17	67	13	19.8

### Intralaboratory reproducibility.

**FIG 6:**
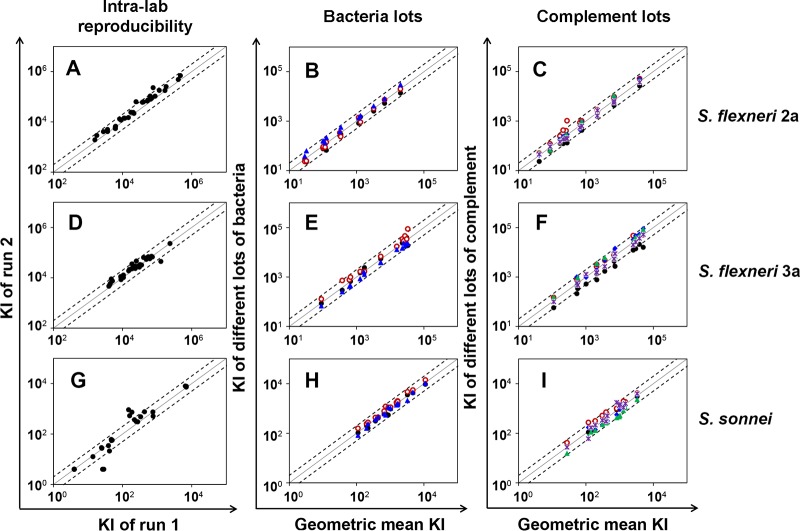
Killing indices (KIs) of run 2 were compared to those of run 1 (left) or KIs of multiple runs to geometric mean KIs (middle and right) to examine intralaboratory reproducibility of SBA for S. flexneri 2a (A, B, and C), S. flexneri 3a (D, E, and F) and S. sonnei (G, H, and I). (A, D, and G) The experiments whose results are shown used the same reagents. (B, E, and H) The experiments whose results are shown used 3 different bacterial stocks. Bacterial stocks were kept frozen at −80°C for various periods (solid black circles, ~3 months old; solid blue triangles, ~1 year old; and solid red circles, ~4 years old). (C, F, and I) The experiments whose results are shown used 5 different complement lots. Five different complement lots from Pel-Freez are shown with different colored symbols. The line of identity (solid line) is shown along with dotted lines indicating 2-fold deviations from identity. The results shown in the middle and right panels suggest that KIs generally differ by less than 2-fold regardless of target bacterium or complement lot and that KIs are offset for all samples tested with each lot of bacteria or complement.

To investigate assay reproducibility, defined as concurrence of results using unknown samples and achieved in several assays conducted on different days, 33 human sera from adult volunteers who were intranasally immunized with S. flexneri 2a invasin complex (invaplex) (22) and later challenged with S. flexneri 2a 2457T were tested twice using the same complement and target bacterial lots (Fig. 6A, D, and G). The sera had high KIs (>10^3^) and yielded highly reproducible results for S. flexneri 2a and S. flexneri 3a (Fig. 6A and D). Only 2 of 33 samples (6%) yielded KIs that differed more than 2-fold for S. flexneri 2a, and only 1 of 33 samples (3%) produced a result deviating more than 2-fold for S. flexneri 3a. For S. sonnei, most (91%) samples had low SBA KIs (<10^3^), and the results were slightly more variable than the results against the two S. flexneri types. And yet, their SBA results were quite comparable, as only five (15%) samples deviated more than 2-fold (Fig. 6G).

Changes in reagents, such as the target bacterium or complement, can influence assay variability. When we investigated the effects of changing biological reagents, we used a new set of serum samples that included specimens with low KIs. When we analyzed the samples with different lots of target bacteria (Fig. 6B, E, and H), the results were quite reproducible, as they varied less than 2-fold. It is worth noting that some bacterial lots were kept frozen for more than 4 years at −80°C. Furthermore, the results with one bacterial lot tended to be higher (or lower) than with the other, and these lot-associated trends could be better visualized when the ratio of the individual KI to the geometric mean of KIs was plotted for each sample (Fig. S4A). Similar to the results for bacterial lots, different complement lots produced results that varied less than 2-fold (Fig. 6C, F, and I). Again, the results with one complement lot tended to be higher than the results for the other for all three serotypes (Fig. 6C, F, and I), and the lot-associated trend can be better observed as shown in Fig. S4B. Thus, this SBA generated reproducible (<2-fold variation) results even with different biological reagents. Furthermore, the variations are reagent lot associated, and they should be even further reducible by analyzing test samples along with a reference serum with an assigned value and then normalizing test sample results with the reference serum result.

10.1128/mSphere.00146-18.4 FIG S4  Individual KI divided by geometric mean KI (*y* axis) was plotted against geometric mean KIs (*x* axis) obtained with the 3 lots of bacterial working stocks (A) or 5 lots of complement (B). Bacterial stocks (indicated by different symbols) were kept frozen at −80°C for various periods (from 3 months to 4 years). The results show that KIs vary generally less than 2-fold regardless of target bacterial or complement lots. Download FIG S4, DOCX file, 0.1 MB. Copyright © 2018 Nahm et al. 2018 Nahm et al. This content is distributed under the terms of the Creative Commons Attribution 4.0 International license.

### Interlaboratory reproducibility.

**FIG 7:**
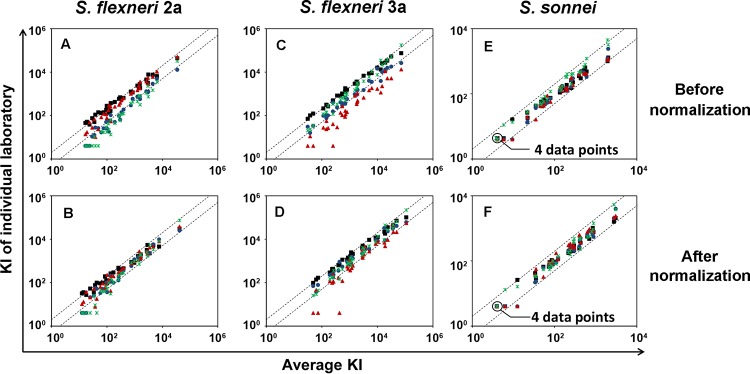
Interlaboratory reproducibility of SBA for the three serotypes, S. flexneri 2a (A and B), S. flexneri 3a (C and D), and S. sonnei (E and F), determined with 32 adult human sera. KIs from individual laboratories are compared against the average KI before (A, C, and E) and after (B, D, and F) normalization. The normalization used Korean QC19 as the reference serum, with assigned KIs of 28,000 for S. flexneri 2a, 19,000 for S. flexneri 3a, and 1,100 for S. sonnei. Average KI shows the geometric means of KIs from the four laboratories (marked with different colors). The dotted lines indicate 2-fold deviations from identity.

Ultimately, a successful assay must be robustly able to produce comparable results in different laboratories. To assess the interlaboratory reproducibility of our new SBA, one central laboratory provided to three other laboratories the complement, bacterial lots, and test samples, along with the assay protocol. The test samples included one human reference serum, MFDS-Ewha PnQC19 from South Korea (abbreviated “Korean QC19” hereinafter), which has assigned SBA values and was used for assay normalization. The test samples were analyzed by the four laboratories for reactivity against S. flexneri 2a, S. flexneri 3a, and S. sonnei. The individual results obtained from the four independent laboratories were compared against the average of results from all four laboratories before and after normalization with Korean QC19 (Fig. 7). Before normalization, it can clearly be seen that one laboratory may produce results lower or higher than those of other laboratories. However, normalization greatly reduced the laboratory-associated variances, and only 14.3% of results deviated from the mean by more than 2-fold. The improved SBA was also compared side-by-side with a previously published SBA developed by the University of Maryland (15). The specimens tested included LPS-specific monoclonal antibodies with high, medium, and low titers and sera from adult human volunteers obtained pre- and postvaccination with live, attenuated S. flexneri 2a strain CVD 1204. A strong agreement was found between the SBA titers obtained by both methods (*r*^2^ = 0.95, *P* < 0.0001) (Fig. S5). Thus, our SBA is simple and robust and produces comparable results when applied by different laboratories. The results were also in agreement with those produced by other laboratories using their own protocols.

10.1128/mSphere.00146-18.5 FIG S5  Data points represent SBA titers obtained using protocols developed at University of Maryland (15) and University of Alabama—Birmingham (described herein) for LPS-specific monoclonal antibodies (squares) and sera from adult volunteers before and 28 days after oral immunization with vaccine strain CVD1204 (open and closed circles, respectively). *R*^2^ = 0.95; *P* < 0.0001. Download FIG S5, DOCX file, 0.04 MB. Copyright © 2018 Nahm et al. 2018 Nahm et al. This content is distributed under the terms of the Creative Commons Attribution 4.0 International license.

### Application of the optimized *Shigella* bactericidal assay.

One of the goals guiding the development of the SBA was the use of the assay in *Shigella* vaccine studies and controlled human infection models (CHIMs) to evaluate the ability of vaccine constructs or bacterial infection to induce functional antibodies capable of killing *Shigella* spp. In a recent phase 1 safety and immunogenicity study that utilized a dose escalation design, serum samples were collected from subjects intranasally immunized on days 0, 14, and 28 with 50 µg of S. flexneri 2a artificial invaplex (ClinicalTrials registration no. NCT02445963; K. A. Clarkson, C. Duplessis, K. R. Turbyfill, C. Porter, R. Gutierrez, M. S. Riddle, T. Lee, H. E. Weerts, S. C. Sumlin, A. Lynen, E. V. Oaks, K. Paolino, G. Fornillos, and R. W. Kaminski, presented at Vaccines for Enteric Diseases 2017, Albufeira, Portugal, 9 to 11 October 2017). Samples collected before immunization and 2 weeks after the third immunization were assayed in the optimized SBA for activity against S. flexneri 2a 2457 T. Additionally, a separate phase 1 study was conducted to determine and verify a dose of S. sonnei strain 53G that safely induced a 60 to 75% attack rate in naive volunteers. S. sonnei-specific SBA titers were determined using serum samples collected before challenge and at various time points postchallenge (ClinicalTrials registration no. NCT02816346; R. W. Frenck, M. Dickey, A. E. Suvarnapunya, L. Chandrasekaran, R. W. Kaminski, M. McNeal, A. Lynen, S. Parker, A. Hoeper, S. Mani, C. Porter, and M. Venkatesan, presented at Vaccines for Enteric Diseases, Albufeira, Portugal, 9 to 11 October 2017). A subset of representative results from each study is outlined in Table 5. Recipients of the S. flexneri 2a invaplex vaccine developed an SBA response that peaked on day 56 (28 days after the third vaccination) with fold increases of 36 to 79 over baseline titers. Similarly, subjects challenged with 1,500 CFU of S. sonnei 53G developed a bactericidal antibody response that peaked 2 weeks after the initial challenge with fold increases over the baseline ranging from 64 to 310. Baseline SBA titers ranged from 1,073 to 2,194 in the S. flexneri 2a assay, whereas the baseline titers were significantly lower in the S. sonnei study (range, 20 to 381). Collectively, these data demonstrate the utility of the SBA in *Shigella* vaccination and CHIM evaluations.

**TABLE 5:** *Shigella* bactericidal responses in subjects immunized with S. flexneri 2a artificial invaplex or orally challenged with S. sonnei 53G in different clinical trials

Vaccine or challenge strain, dose (type of study; ClinicalTrials registration no.)	Subject ID^a^	Day of study	KI	Fold increase over baseline
S. flexneri 2a artificial invaplex, 50 µg (vaccine trial; NCT02445963)	30	0	1,073	
		56	84,827	79
	35	0	1,675	
		56	100,552	60
	36	0	2,194	
		56	78,378	36
S. sonnei 53G, 1,500 CFU (CHIM^b^; NCT02816346)	134	0	20	
		14	6,107	305
	135	0	20	
		14	6,202	310
	137	0	381	
		14	24,246	64

a ID, identification no.

b CHIM, controlled human infection model.

## DISCUSSION

The exact immunological correlates of protection against shigellosis remain to be defined. Based on epidemiological studies and challenge/rechallenge experiments in humans and nonhuman primates, protection against *Shigella* is known to be serotype specific (8, 24). Since LPS, and more precisely, the O polysaccharide of LPS, determines the serotype specificity, immune responses directed to LPS are likely involved with protection. The role of LPS-specific antibodies and the extent to which they contribute to protection are also largely unknown but presumably involve a functional attribute, such as opsonophagocytosis and/or complement activation. Antibodies with shigellacidal activity have been detected in sera of naturally infected individuals by using traditional complement-mediated killing assays (16, 25). These assays, however, are difficult to perform and time consuming, and the results can be highly variable and difficult to compare. Therefore, we have successfully developed and qualified a simple, high-throughput assay to measure *Shigella* SBA activity.

Improvements that greatly simplified our SBA platform included the use of (i) cryopreserved target strains, which greatly reduced time and assay variability by avoiding the need to grow fresh organisms the day of the assay, and (ii) a microculture system, which drastically reduced the number of agar plates needed for colony counting and enabled the use of a processing system, originally developed for the pneumococcal multiplexed opsonophagocytic killing assay (MOPA) (17), which automatically converts colony counts into bacterial KIs. These features allow for the processing of large numbers of specimens and generation of data in a high-throughput manner.

Using the SBA configuration described herein, 25 to 50 specimens can be tested per day by a single operator. The analytical throughput of the described *Shigella* SBA is comparable to that of another high-throughput assay described recently by Necchi et al. (14). The Necchi assay measures ATP released by the killed bacteria and requires specialized equipment for ATP measurement (14). In contrast, our SBA requires no special equipment and uses readily available bacterial strains that are in the public domain, clinically relevant, and used in CHIM studies. For instance, the colony counting system requires only a conventional digital camera and free software (26). Thus, our SBA is simple enough to be adopted by laboratories around the world.

The characterization of the analytical performance of our SBA demonstrated adequate sensitivity, linearity, precision, and reproducibility (robustness). For our studies, most sera from healthy adults or immunized animal sera (data not shown) could be prediluted more than 100-fold. High assay sensitivity is important when analyzing small specimen volumes from children, who are a key target population for a *Shigella* vaccine. When the assay linearity was tested, the observed values matched the expected values for high KIs (>40 KI) but were slightly higher (less than 2-fold) than expected for KIs of less than 40. The observed nonlinearity may not be significant, since a 2-fold variation is generally accepted in OPAs and other functional assays (23). In terms of precision, the median intra- and interassay CVs were 13% and 28%, respectively. Furthermore, the SBA is quite reproducible, since most KIs could be replicated within a 2-fold variation even when different lots of complement and target bacteria were used.

To characterize our SBA, we used sera from adults who received LPS containing *Shigella* vaccines or who have natural immunity to *Shigella*, which is mostly mediated by antibodies to O antigen (8–11). For vaccine candidates based on antigens other than LPS, the assay would have to be adapted and reevaluated using target strains expressing the antigen in question, and as long as bactericidal activity is induced, the traditional SBA format described here should be able to detect it.

A factor found important for high intra- and interlaboratory comparability was normalization of results with a reference serum. Since there is no *Shigella* reference serum, we used Korean QC19, which has relatively high KIs to all three serotypes studied here and is being made available for the research community worldwide. The impact of normalization found in our work is consistent with experiences with pneumococcal OPAs. Normalization with reference serum 007sp significantly reduced the variability of OPA results from six different laboratories (27). While Korean QC19 can be used as a provisional reference serum, a *Shigella* reference serum should be developed as soon as possible.

Most assays that are designed to measure the functional capacity of antibodies have not been examined for interlaboratory reproducibility. And yet, the demonstration of interlaboratory reproducibility is critical to confirm the adequate performance of any assay used for vaccine studies and for establishing correlates of protection. Thus, the most exciting finding of this study is the demonstration of a very high degree of interlaboratory comparability. For this study, the UAB laboratory provided other laboratories with the three key reagents—target bacteria, complement, and test sera, as well as colony counting software and the assay protocol. Providing reagents was not difficult because aliquots of target bacteria and complement can be cryopreserved for long periods. Two laboratories had extensive experience performing *Shigella* SBAs, while the third had minimal experience and training. Thus, other laboratories may be able to adopt the SBA described with support from a central laboratory. The availability of reagents and a central reference laboratory should facilitate assay harmonization, vaccine evaluation, and vaccine licensure by regulatory agencies (30).

In addition to describing the development, optimization, and interlaboratory evaluation of a *Shigella* SBA, the utility of the assay in the context of clinical studies was investigated. Serum samples from a phase 1 *Shigella* vaccine study and from a phase 1 CHIM study were tested using the optimized S. flexneri 2a and S. sonnei-specific SBA protocols, respectively, to determine the presence of functional antibodies following vaccination or challenge. The high SBA titers obtained demonstrate not only the capacity of the assay in detecting bactericidal responses to multiple *Shigella* serotypes but also that these antibodies are induced both by infection with virulent organisms and by vaccination with LPS-containing subunit vaccine candidates. A more complete analysis of the bactericidal antibodies induced in these two studies is under way. The described SBA has been adopted by several research groups evaluating *Shigella* vaccine candidates and will be used in upcoming *Shigella* CHIM studies. Serum bactericidal antibodies, along with other immunological outcomes, are being explored as potential immune correlates/surrogates of protection.

## MATERIALS AND METHODS

### Serum samples, MAbs, and purified LPS.

Ten archived, anonymous sera and plasma which were collected between 1994 and 1999 from healthy adults at Washington University in St. Louis and the University of Rochester were used. Three archived, anonymous sera from three children were obtained from F. Russell in Australia. Thirty-three anonymized serum samples were collected from 11 volunteers who were intranasally immunized on days 0, 14, and 28 with either S. flexneri 2a invaplex or normal saline (22) and orally challenged on day 60 with 800 CFU of S. flexneri 2a 2457T (ClinicalTrials registration no. NCT00485134). The use of these anonymous samples was classified as non-human subject research by the UAB IRB (protocol number N150115001). Ten commercially available human sera (LOB1 to -10) were purchased from Innovative Research, Inc. (Novi, MI). A human serum pool, 007sp, was acquired from the U.S. FDA (20). Six human sera (MFDS-Ewha PnQC3, -9, -10, -19, -22, and -29) were acquired from Kyung-Hyo Kim in Ewha Womans University School of Medicine, South Korea. These sera are referred to herein as Korean QC sera and were collected from healthy volunteers who were immunized with the 23-valent polysaccharide vaccine PPV23.

Six LPS-specific mouse MAbs (Hflex2a1, Hflex2a4, Hflex3a2, Hflex3a5, Hsoni1, and Hsoni5) were previously described (28). Hflex2a1 and Hflex2a2 are specific for S. flexneri 2a LPS, Hflex3a2 and Hflex3a5 are specific for S. flexneri 3a LPS, and Hsoni1 and Hsoni5 are specific for S. sonnei LPS. LPS was purified from S. flexneri 2a 2457T, S. flexneri 3a J17B, and S. sonnei Moseley by the Westphal procedure (29), using hot phenol-water extraction.

### SBA protocol.

SBA was performed by mixing 10 µl of target bacteria (~10^5^ CFU/ml), 50 µl of diluted baby rabbit complement (12.5% final concentration), and 20 µl of a heat-inactivated, serially diluted test sample in a well of a round-bottom microtiter plate. Control wells had bacteria, rabbit complement, and buffer only; no test sample was added to these wells. Target bacteria were prepared by thawing a frozen aliquot of the target bacteria and diluting in assay buffer to the desired bacterial density. The reaction mixture was incubated for 2 h at 37°C in room air, and then 10 µl from each well was spotted on LB agar. (The volume was chosen to have about 120 CFU in the counting area on the agar plate.) The agar plates were incubated overnight (~16 h) at 29°C for S. flexneri 2a and S. flexneri 3a and at 26°C for S. sonnei. The agar plates were overlaid with agar containing 100 µg/ml triphenyl tetrazolium chloride (TTC) and 0.1% sodium azide and then incubated for an additional 2 h at 37°C for color development. TTC is converted to a red 1,3,5-triphenylformazan due to the activity of dehydrogenases in metabolically active bacteria, creating significant contrast between the agar plate and the bacterial colonies. Photographs of the agar plates were obtained with a digital camera.

### Calculations and statistical evaluations.

Digital images were analyzed with a computer program, NIST’s Integrated Colony Enumerator (NICE) (26), to determine the number of colonies in each spot. The colony numbers were then converted to the SBA killing index (KI) as detailed below. First, we determined the average number of CFUs per spot of control wells to obtain the target bacterial number. Then, the dilution of a sample that kills 50% of the target bacteria was defined as the KI. In practice, KI was determined with a data processing pipeline called Opsotiter. A detailed protocol is available at http://www.vaccine.uab.edu/SBAProtocol.pdf.

The KIs were normalized using the following formula: normalized KI of sample = measured KI of sample × (KI assigned to Korean QC19/geometric mean of measured KI of Korean QC19).

The KIs were log_10_ transformed for statistical analysis. Pearson’s *R*^2^ values were calculated to evaluate the correlation between the KIs of human sera against multiple strains belonging to the three *Shigella* serotypes used in the SBA. Comparisons of assay precision and reproducibility were evaluated by calculating coefficients of variation.

The nonspecific killing (NSK) percentage was calculated using the following formula: [(CFU of control without complement − CFU of control with complement)/CFU of control without complement] × 100 = NKS.

Korean QC19 was assigned to have KIs of 28,000 for S. flexneri 2a, 19,000 for S. flexneri 3a, and 1,100 for S. sonnei. Korean QC19 was analyzed three times in each run, and the geometric mean value was used for normalization.

### Laboratories that participated in interlaboratory comparison.

The participating laboratories were M. Nahm’s laboratory at University of Alabama at Birmingham in Birmingham, AL, R. Kaminski’s and M. Venkatesan's laboratory at Walter Reed Army Institute of Research in Silver Spring, MD, and M. Pasetti’s laboratory at University of Maryland in Baltimore, MD.
