# Supplementary figures and images for: Development, Interlaboratory Evaluations, and Application of a Simple, High-Throughput Shigella Serum Bactericidal Assay
Source: mSphere. 2018 Jun 13;3(3):e00146-18. doi: 10.1128/mSphere.00146-18 (PMC6001606; doi:10.1128/mSphere.00146-18)

**Supplementary Figure 1:**

| 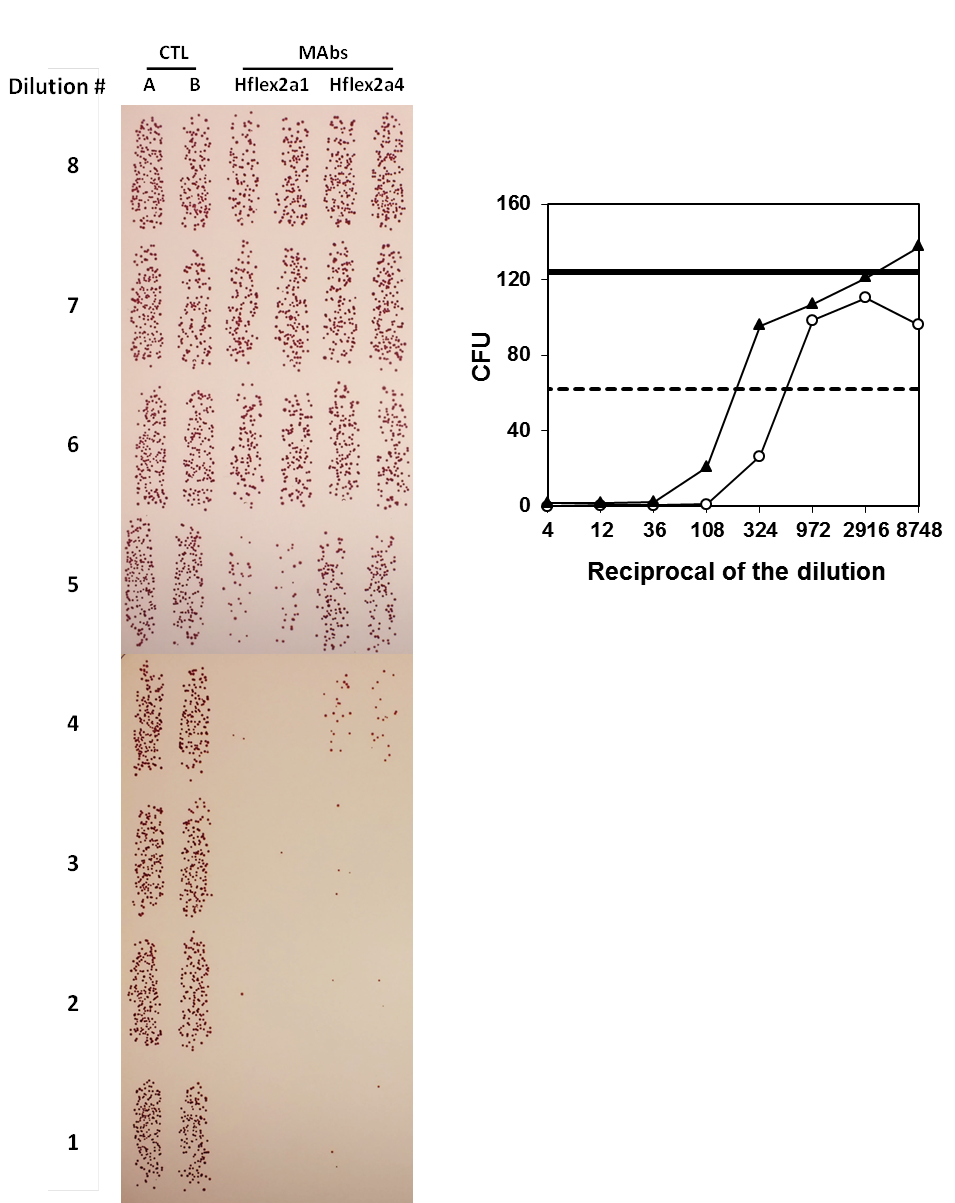 |
| --- |
|  |

Supplement: FIG S1 [file sph003182554sf1.docx]

**Supplementary Figure 2:**

| 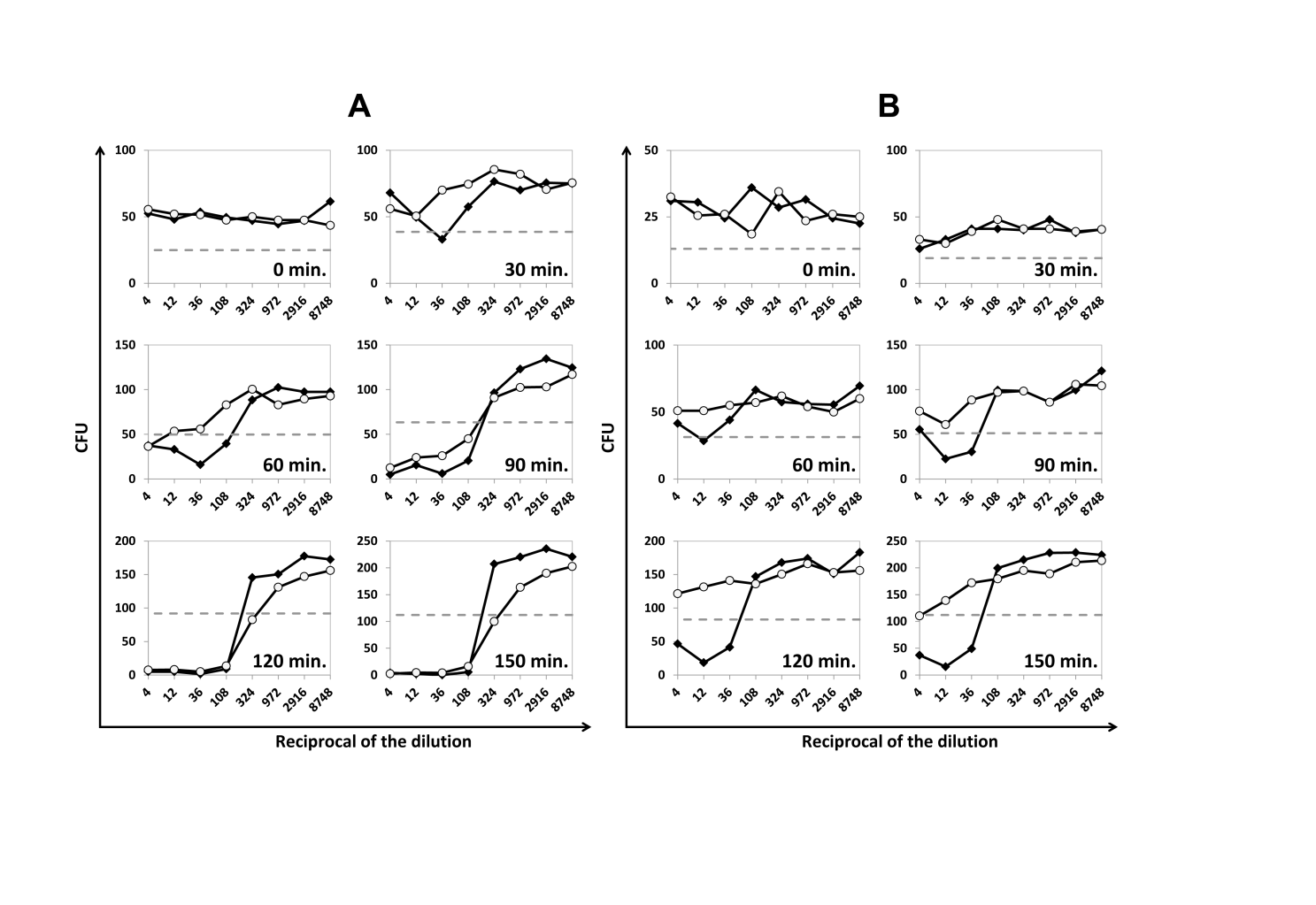 |
| --- |

Supplement: FIG S2 [file sph003182554sf2.docx]

| **2a vs x** | **3a vs x** | **3a vs x** | **sonnei vs x** | **sonnei vs x** |
| --- | --- | --- | --- | --- |
| **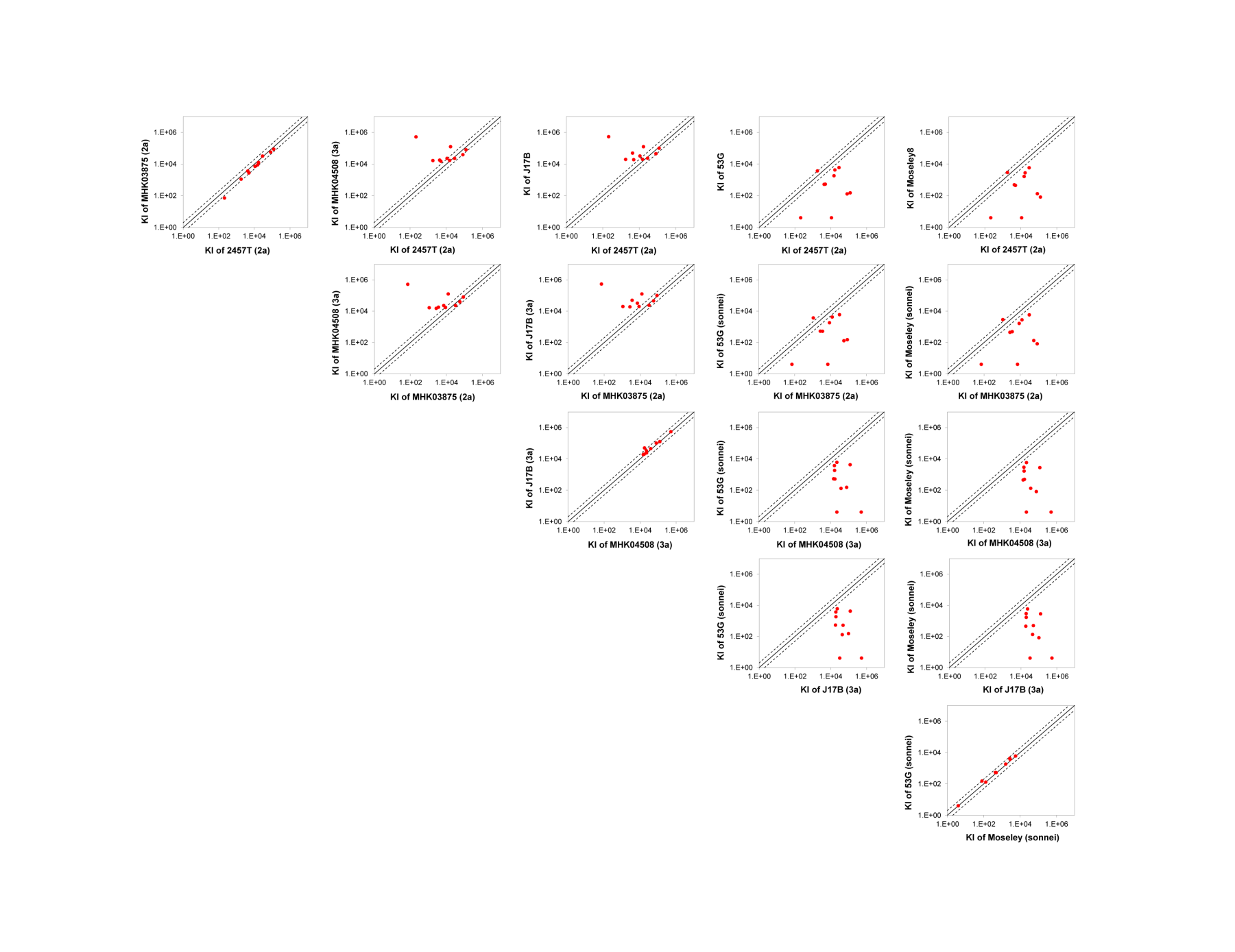** | | | | |
|
|
|
|

**Supplementary Figure S3**

Supplement: FIG S3 [file sph003182554sf3.docx]
