# Supplementary material for: Development, Interlaboratory Evaluations, and Application of a Simple, High-Throughput Shigella Serum Bactericidal Assay
Source: mSphere. 2018 Jun 13;3(3):e00146-18. doi: 10.1128/mSphere.00146-18 (PMC6001606; doi:10.1128/mSphere.00146-18)
Supplement: FIG S4 [file sph003182554sf4.docx]

**Supplementary Figure 4:**

| **A.Bacteria working stock lots**  **Individual KI/Geometric mean KI** | **B.Complement lots** |
| --- | --- |
|  |  |
|  |  |
| **Sample number (Geometric mean KI low-->high)** |  |
