# Supplementary material for: Development, Interlaboratory Evaluations, and Application of a Simple, High-Throughput Shigella Serum Bactericidal Assay
Source: mSphere. 2018 Jun 13;3(3):e00146-18. doi: 10.1128/mSphere.00146-18 (PMC6001606; doi:10.1128/mSphere.00146-18)
Supplement: FIG S5 [file sph003182554sf5.docx]

**Supplementary Figure 5**
